# Long‐term caloric restriction ameliorates deleterious effects of aging on white and brown adipose tissue plasticity

**DOI:** 10.1111/acel.12948

**Published:** 2019-03-28

**Authors:** Patricia Corrales, Yurena Vivas, Adriana Izquierdo‐Lahuerta, Daniel Horrillo, Patricia Seoane‐Collazo, Ismael Velasco, Lucia Torres, Yamila Lopez, Carmen Martínez, Miguel López, Manuel Ros, Maria Jesus Obregon, Gema Medina‐Gomez

**Affiliations:** ^1^ Department of Basic Sciences of Health, Area of Biochemistry and Molecular Biology Universidad Rey Juan Carlos Alcorcon‐Madrid Spain; ^2^ NeurObesity Group, Department of Physiology CIMUS, University of Santiago de Compostela‐Instituto de Investigación Sanitaria Santiago de Compostela Spain; ^3^ CIBER Fisiopatología de la Obesidad y Nutrición (CIBERobn) Madrid Spain; ^4^ MEMORISM Research Unit Institute of Biomedical Research "Alberto Sols" (CSIC), University Rey Juan Carlos Madrid Spain; ^5^ Endocrine and Nervous System Pathophysiology Instituto de Investigaciones Biomédicas (IIB), Centro Mixto CSIC‐UAM (Consejo Superior Investigaciones Científicas and Universidad Autonoma de Madrid) Madrid Spain

**Keywords:** adipose tissue, aging, caloric restriction, fibro‐inflammation, insulin resistance, thyroid hormone

## Abstract

Age‐related increased adiposity is an important contributory factor in the development of insulin resistance (IR) and is associated with metabolic defects. Caloric restriction (CR) is known to induce weight loss and to decrease adiposity while preventing metabolic risk factors. Here, we show that moderate 20% CR delays early deleterious effects of aging on white and brown adipose tissue (WAT and BAT, respectively) function and improves peripheral IR. To elucidate the role of CR in delaying early signs of aging, young (3 months), middle‐aged (12 months), and old (20 months) mice fed al libitum and middle‐aged and old mice subjected to early‐onset CR were used. We show that impaired plasticity of subcutaneous WAT (scWAT) contributes to IR, which is already evident in middle‐aged mice. Moreover, alteration of thyroid axis status with age is an important factor contributing to BAT dysfunction in middle‐aged animals. Both defects in WAT and BAT/beige cells are ameliorated by CR. Accordingly, CR attenuated the age‐related decline in scWAT function and decreased the extent of fibro‐inflammation. Furthermore, CR promoted scWAT browning. In brief, our study identifies the contribution of scWAT impairment to age‐associated metabolic dysfunction and identifies browning in response to food restriction, as a potential therapeutic strategy to prevent the adverse metabolic effects in middle‐aged animals.

## 1 INTRODUCTION

Aging is associated with an increased risk of metabolic disorders such as obesity, insulin resistance (IR), and other manifestations of metabolic syndrome in both humans and rodents. In parallel with these alterations, a low grade of inflammation has also been described in several tissues associated with aging (DeNino et al., 2001; Hughes et al., 2004). Progressiveness is one of the characteristics of the aging process and according with this concept, aging begins right after maturity and the deleterious effects of physiological functions progress and worsen along the lifespan of the organism. Aging is typically associated with increased adiposity and redistribution of adipose tissue (AT), characterized by a loss of subcutaneous adipose depot mass and a gain of fat in the abdominal visceral compartment (Carrascosa et al., 2011; Redman et al., 2018).

Caloric restriction (CR) is the most efficient intervention to delay the deleterious effects of age‐related metabolic diseases (Colman et al., 2014; Redman et al., Carobbio, S., Pellegrinelli, V., & Vidal‐Puig, A.(2017). Adipose^2018^; Walker, Houthoofd, Vanfleteren, & Gems, 2005). Previous studies in several animal models have shown that CR has physiological effects on lifespan, and reduces body weight and glucose and insulin serum levels (Anderson, Shanmuganayagam, & Weindruch, 2009; Fabbiano et al., 2016; Mitchell et al., 2016). Whether CR interventions in humans slow aging is not yet known. Accumulating data indicate that moderate CR with adequate nutrition has numerous beneficial effects against obesity, diabetes, inflammation, and cardiovascular diseases (Fontana et al., 2016; Redman et al., 2018). However, the mechanisms involved in the amelioration of aging effects by CR are not well understood. Accretion of AT has been related to the development of age‐associated metabolic alterations such as IR. Moreover, reduction of adiposity by CR (Escriva et al., 2007) or fat removal (Gabriely et al., 2002) have demonstrated to ameliorate age‐associated IR. The improvement of the metabolic status achieved by CR may well be due, at least in part, to the decreased adiposity, as suggested by other authors (Basu et al., 2003; Ferrannini et al., 1996). Furthermore, increased adiposity by hypertrophy and/or hyperplasia has been demonstrated to increase macrophage infiltration. This circumstance, together with changes in adipocyte physiology that includes hypoxia, reticulum, and oxidative stress, leads to an inflammatory state which is a key factor in the AT expandability capacity (Carobbio, Pellegrinelli, & Vidal‐Puig, 2017). Nevertheless, AT is a complex organ with different localizations and functions beyond its traditional role as a fat storage unit.

A complete understanding of CR effects on AT biology requires the elucidation of whether these effects are preferentially mediated by white AT (WAT) and/or brown AT (BAT), the contribution of specific WAT depots, and the relevance of differentiation/trans‐differentiation to beige AT (Frontini et al., 2013; Maurizi et al., 2017). WAT also has an important endocrine role by secreting different peptide hormones (adipokines) including adiponectin, which regulates insulin sensitivity, as well as glucose and energy homeostasis. In contrast to WAT, BAT plays a central role in energy expenditure *via* expression of uncoupling protein 1 (UCP‐1) (Cannon & Nedergaard, 2009). BAT is the major site for both cold‐ and diet‐induced thermogenesis, and its atrophy has been observed in obese and older individuals in association with increased visceral fat and hyperglycemia (Cypess et al., 2009). Consequently, defective WAT and BAT function may exacerbate the development of metabolic complications of obesity/aging.

Here, we aimed to investigate whether the plasticity of the WAT and BAT depots (hypertrophy and/or hyperplasia, extracellular matrix remodeling, inflammation, and browning or whitening capacity) is differentially affected at middle age, and whether moderate CR results in beneficial metabolic effects regulating the functionality of these AT depots. We show that several metabolic alterations of old animals are already being developed in middle‐aged animals. These alterations include development of IR, altered WAT and BAT plasticity, as well as alteration of thyroid axis status, which can be mitigated, at least to some extent, by moderate and long‐term CR.

## 2 RESULTS

### 2.1 Aging is associated with insulin resistance

**Figure 1:**
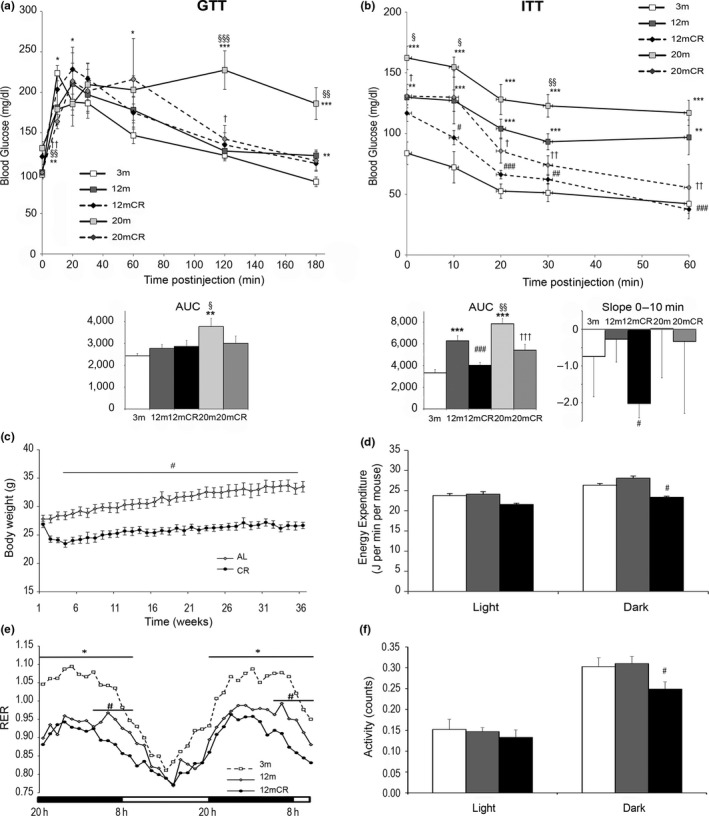
Effect of aging and caloric restriction on body weight, energy expenditure, and insulin sensitivity. (a) GTT and (b) ITT curves with their respective AUC from 3‐m, 12‐m, 12mCR, 20‐m, and 20mCR mice. Slopes from 0 to 10 m in ITT are also represented in (b). (c) Evolution of body weight in 12‐month‐old mice fed ad libitum (12 m) or 20% CR (12mCR). (d–f) Indirect calorimetry was measured every hour: (d) energy expenditure (J/min/mouse), (e) respiratory exchange ratio (RER), and (e) locomotor activity (counts). Data are expressed as mean ± *SEM* (a, b: *n* = 7–9 animals/group; c: *n* = 11–24 animals/group; d–f: *n* = 3–9 animals/group). * *p* < 0.05, ** *p* < 0.01, *** *p* < 0.01 12 m or 20 m vs. 3 m; § *p* < 0.05, §§ *p* < 0.01, §§§ *p* < 0.001 20 m vs. 12 m; # *p* < 0.05, ## *p* < 0.01, ### *p* < 0.001 12mCR vs. 12 m; † *p* < 0.05 20mCR vs. 20 m

First, we investigated the effects of aging on body weight (BW), glucose homeostasis, and insulin response (Table 1 and Figure 1) in male 129S2/SvPasCrl mice fed ad libitum (AL) at 3, 12, and 20 months of age, defined as young, middle‐aged, and old mice, respectively. BW and fat depots mass significantly increased in middle‐aged compared to young mice. Older animals showed a significant decrease in these weights respect to 12 m. In a similar way, pancreas showed a weight decline in 20‐m mice. Nevertheless, kidney and gastrocnemius exhibited a higher weight in 20‐m than in 12‐m animals.

**Table 1:** Body weight, body mass distribution, and serum parameters measured in 3‐m, 12‐m, 12mCR, 20‐m, and 20mCR mice

	3 m	12 m	12mCR	20 m	20mCR
Body weight (g)	22.36 ± 0.42	34.10 ± 0.91***	27.25 ± 0.59###	25.39 ± 0.72**§§§	20.67 ± 0.33†
scWAT (g)	0.14 ± 0.01	0.33 ± 0.007**	0.22 ± 0.02#	0.23 ± 0.02**§§	0.30 ± 0.02
eWAT (g)	0.18 ± 0.02	0.48 ± 0.02**	0.27 ± 0.03##	0.26 ± 0.05§§	0.14 ± 0.03
BAT (g)	0.09 ± 0.006	0.18 ± 0.02**	0.11 ± 0.01#	0.07 ± 0.003§§§	0.07 ± 0.004
Liver (g)	1.05 ± 0.07	1.37 ± 0.08*	1.07 ± 0.04##	1.09 ± 0.11	0.76 ± 0.09
Pancreas (g)	0.22 ± 0.02	0.30 ± 0.01*	0.29 ± 0.02	0.17 ± 0.009§§	0.14 ± 0.01
Kidney (g)	0.14 ± 0.01	0.24 ± 0.005***	0.19 ± 0.003###	0.36 ± 0.01***§§§	0.29 ± 0.0003†
Soleus muscle (mg)	7.3 ± 1.64	6.46 ± 0.79	10.92 ± 1.70	8.3 ± 1.1	8.7 ± 1.14
Gastrocnemius muscle (g)	0.09 ± 0.005	0.13 ± 0.005**	0.10 ± 0.008#	0.19 ± 0.006**§§	0.19 ± 0.007
Fasting glucose (mg/dl)	98.25 ± 5.40	100.80 ± 4.07	120.4 ± 9.66#	130.8 ± 8.1**§§	100.8 ± 6.5†
Fasting insulin (µg/L)	0.22 ± 0.11	0.61 ± 0.06*	0.15 ± 0.05#	0.62 ± 0.20	0.49 ± 0.10
Nonfasting glucose (mg/dl)	133.20 ± 6.46	129.00 ± 5.56	108.98 ± 5.46#	122.00 ± 10.19	105.6 ± 5.23
Nonfasting insulin (µg/L)	0.50 ± 0.08	1.07 ± 0.13**	0.72 ± 0.14	0.90 ± 0.26	0.30 ± 0.03
HOMA_IR_	1.51 ± 0.34	4.38 ± 0.15**	1.29 ± 0.20###	5.74 ± 0.94**	5.61 ± 1.31
Nonfasting leptin (ng/ml)	4.82 ± 0.83	7.97 ± 2.02	1.91 ± 0.24#	2.84 ± 0.53	2.77 ± 0.57
Nonfasting MCP−1 (pg/ml)	77.43 ± 5.85	200.70 ± 52.74*	67.49 ± 17.59#	123.20 ± 10.78**	80.95 ± 20.94
Nonfasting adiponectin (µg/ml)	13.40 ± 1.08	5.57 ± 0.47*	13.61 ± 2.53#	‐	‐
Serum thyroxine (T4) (ng/ml)	45.5 ± 3.8	31.6 ± 4.4	36.9 ± 4.1	‐	‐
Serum triiodothyronine (T3) (ng/ml)	0.12 ± 0.03	0.09 ± 0.02*	0.19 ± 0.04#	‐	‐
Daily food intake (g)	3.92 ± 0.12	3.96 ± 0.10	3.17 ± 0.02	‐	‐
Daily food intake relative to BW	0.145 ± 0.004	0.113 ± 0.002*	0.117 ± 0.0007	‐	‐

Note

Data are represented as average ± *SEM* (*n* = 3–10 animals/group).

*p < 0.05,**p < 0.01,***p < 0.01 12 m or 20 m vs. 3 m;^§^ *p* < 0.05,^§§^ *p* < 0.01, ^§§§^ *p* < 0.001 20 m vs. 12 m;^#^ *p* < 0.05,^##^ *p* < 0.01,^###^ *p* < 0.001 12mCR vs. 12 m;^†^ *p* < 0.05 20mCR vs. 20 m.

Although no significant differences were found in nonfasting glucose levels among mice from different ages, fasting glucose levels significantly increased in older mice respect to both young and middle‐aged mice. Nonfasting and fasting insulin levels increased from 3 to 12 m and maintained high thereafter (Table 1).

Oral glucose tolerance test (GTT) did not present significant differences between young and middle‐aged animals, but 20‐m mice showed a significant glucose intolerance compared to 3‐m and 12‐m animals (Figure 1a). When the decrease of glycemia in response to insulin was plotted in the ITT, a slight but not significant decrease in the slope during the first 10 min, as an index of insulin responsiveness, was observed in both 12‐m and 20‐m animals (Figure 1b). Nevertheless, the AUC data showed significant increases in 12‐m and 20‐m animals respect to 3‐m control mice. These data together with the significant increase of the HOMA_IR_ index in 12‐m animals suggest that a state of IR is already present in middle‐aged animals (Table 1). Immunohistochemistry data from pancreas (Supporting Information Figure S1) showed that 20‐m mice presented an increased islet size, probably as a compensatory response to ameliorate the state of IR but decreased an insulin content. This, together with the significant increase of the AUC in the GTT and the significant increase of fasting glucose concentration, pointed to a worsening of glucose homeostasis in 20‐m animals which seems to be in an open diabetic state.

### 2.2 Long‐term CR improves age‐associated insulin resistance

CR did not modify GTT in middle‐aged animals. Twenty‐month‐old mice after CR (20mCR) showed a decrease of the AUC, but this change did not reach significance compared to 20‐m mice. In contrast, ITT showed that CR significantly decreased the AUC in both 12‐m and 20‐m animals, suggesting that CR ameliorates IR at both ages (Figure 1b and Supporting Information Figure S1a). The slope of the decline of glycemia during the first 10 min (Figure 1b) showed an increase in both 12mCR and 20mCR animals. Nevertheless, this increase reached significance only in 12mCR mice. HOMA_IR_ data (Table 1) also confirmed a solid recovery of the IR in 12mCR but not in 20mCR animals. Accordingly, the fact that 20‐m mice showed an altered glucose control, as deduced from the increased fasting glycemia and GTT data, as well as signs of pancreatic alterations (Supporting Information Figure S1b), led us to further evaluate the effects of 20% CR from 3 to 12 months of age (12mCR) before metabolic alterations worsen and reach irreversibility on several aspects related to glucose and energy homeostasis.

Whereas *AL*‐fed animals progressively increased their BW over the study period of 12 months, animals subjected to CR presented an initial drop in BW, and this was followed by a slow recovery of BW gain that remained significantly lower than that of *AL*‐fed animals (Figure 1c; Table 1). Accordingly, all tissues analyzed except muscles showed a decreased mass. No differences in daily food intake were found between 3‐m and 12‐m animals but when it was calculated in relation to the BW, this parameter decreased in 12‐m mice (Table 1). Similar data were obtained in 20‐m mice after CR.

Adiponectin levels, which are associated with insulin sensitivity, were significantly lower in 12 m than in 3 m, whereas they were similar between 12mCR and 3‐m mice. Serum leptin concentration increased with aging and CR lowered this parameter to values below those detected in 3‐m animals (Table 1). A decrease of serum MCP‐1 levels in 12mCR was also detected.

To further investigate the effects of CR on metabolic control in middle‐aged animals, we evaluated energy balance by indirect calorimetry (Figure 1d–f). Energy expenditure was not different between 3‐m and 12‐m mice but was significantly lower in their active cycle in 12mCR animals (Figure 1d). The RER was significantly lower in 12‐m than in 3‐m animals during the active cycle, suggesting an increase in the use of fatty acids as the primary substrate for oxidative metabolism (Figure 1e). Interestingly, 12mCR mice exhibited two distinct phases of fuel selection when compared with 12 m. In the first phase, close to the beginning of the dark cycle, when food was administrated, RER was similar between 12mCR and 12‐m mice. However, in the second phase, after the food allotment was finished, RER fell rapidly in 12mCR (close to a value of 0.7) and was significantly lower than 12 m, indicating an almost exclusive use of fat for oxidation in these mice. Finally, while no changes in locomotor activity were detected between 3 and 12 m, it was significantly lower in 12mCR than in equivalent *AL*‐fed mice in the dark period (Figure 1f).

### 2.3 Long‐term CR ameliorates initial age‐associated alterations in scWAT plasticity, inflammation, and fibrosis detected in middle‐aged mice

**Figure 2:**
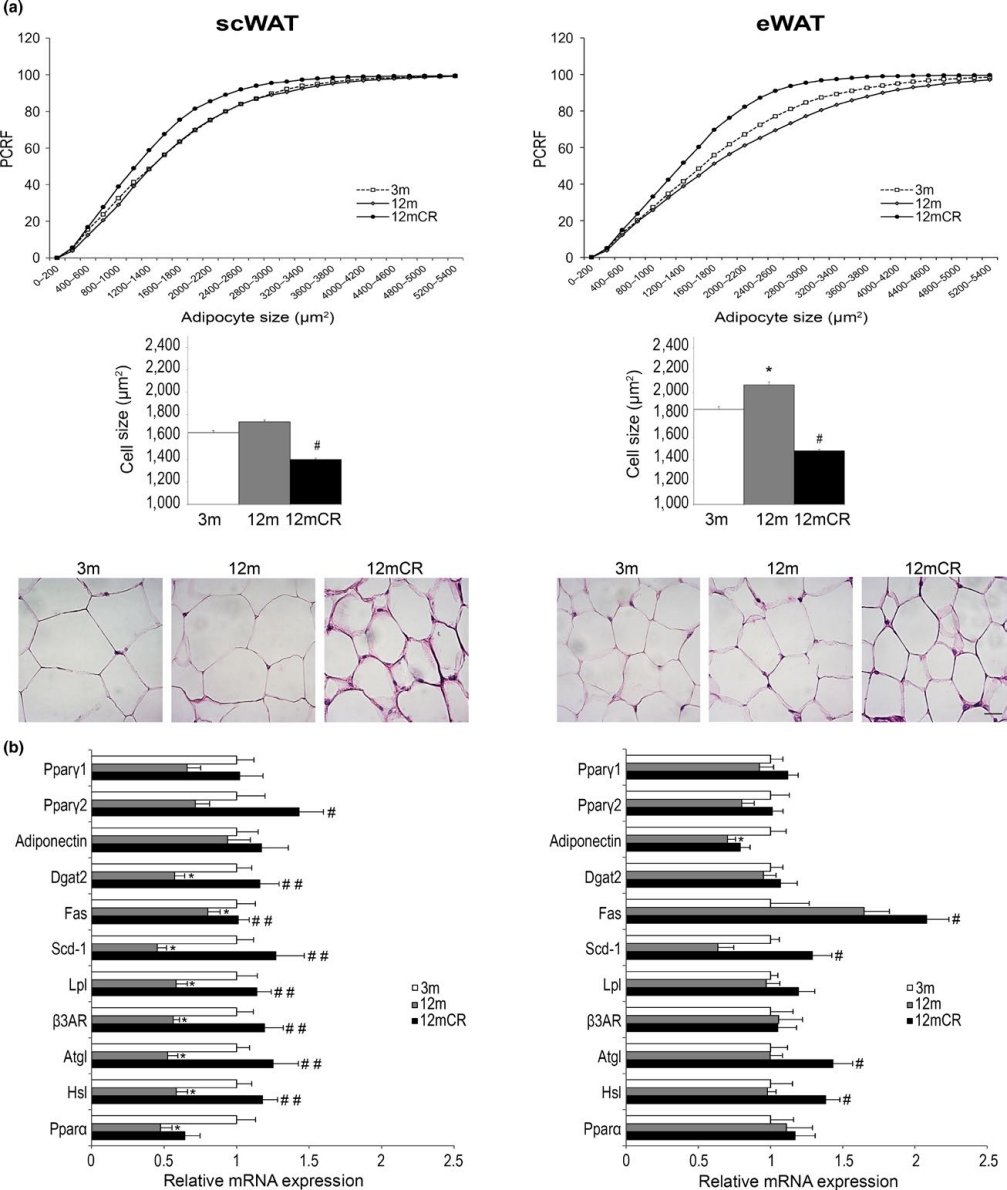
Morphology of adipocytes and expression of adipose markers in subcutaneous and epididymal WAT during aging. (a) Representative images, percent relative cumulative frequency (PRCF) of adipocytes, total adipocyte cell size, and representative images (hematoxylin‐eosin‐stained histological sections (magnification 40×, scale bar = 20μm)) from subcutaneous WAT (scWAT) and epididymal WAT (eWAT). (b) mRNA levels of adipogenic markers and lipid metabolism genes in scWAT and eWAT. All data are expressed as mean ± *SEM* (A, C: *n* = 7–9 animals/group). * *p* < 0.05, 12 m vs. 3 m; # *p* < 0.05, ## *p* < 0.01, 12mCR vs. 12 m

Next, we investigated the potential role of different WAT depots and their functional plasticity in peripheral IR in middle‐aged mice. Examination of scWAT from 3‐m and 12‐m mice revealed no differences in the distribution of adipocyte sizes (Figure 2a), suggesting a poor expandability of subcutaneous adipocytes from 3 to 12 m. As expected, CR brought about a shift of the curve demonstrating a decreased adipocyte size in scWAT. On the other hand, relevant genes for WAT function and lipid metabolism such as *Dgat2*, *Fas, Scd‐1, Lpl, Atgl, Hsl, and Pparα* (Figure 2b) were expressed in scWAT at significantly lower levels in 12‐m than in 3‐m mice, whereas their expression in 12mCR animals was comparable to that of 3‐m. Nevertheless, the mRNA expression of the adipogenic genes *Pparγ1* and *Pparγ2* was comparable between 3‐m and 12‐m mice (Figure 2b), and this was confirmed at the protein level (Supporting Information Figure S2a). Moreover, mitochondrial biogenesis and oxidative capacity of scWAT were also evaluated by means of mRNA expression of *Pgc1*, *Cpt1, *and several OXPHO genes. As shown in Supporting Information Figure S2b, expression of several of these genes decreased in 12‐m respect to 3‐m mice while CR showed a tendency to increase these parameters.

In contrast to scWAT, eWAT showed an increase in the number of larger adipocytes in 12‐m mice compared to 3‐m mice, suggesting that these adipocytes are prone to expansion at this age, and hence accumulate lipids. The effect of CR was a reduction in the adipocyte size again (Figure 2a). Moreover, with the exception of adiponectin, no changes in the expression of the analyzed genes were found between 3‐m and 12‐m mice in eWAT. Similar to the results in scWAT, CR significantly increased the expression of several genes involved in lipid metabolism, including *Fas*, *Scd1, Atgl,* and *Hsl*, and increased the protein expression of PPARγ2 (Figure 2b; Supporting Information Figure S2a). In contrast to scWAT, the expression of the above‐mentioned genes involved in the mitochondrial biogenesis and oxidative capacity of eWAT did not show statistical differences among groups (Supporting Information Figure S2b).

**Figure 3:**
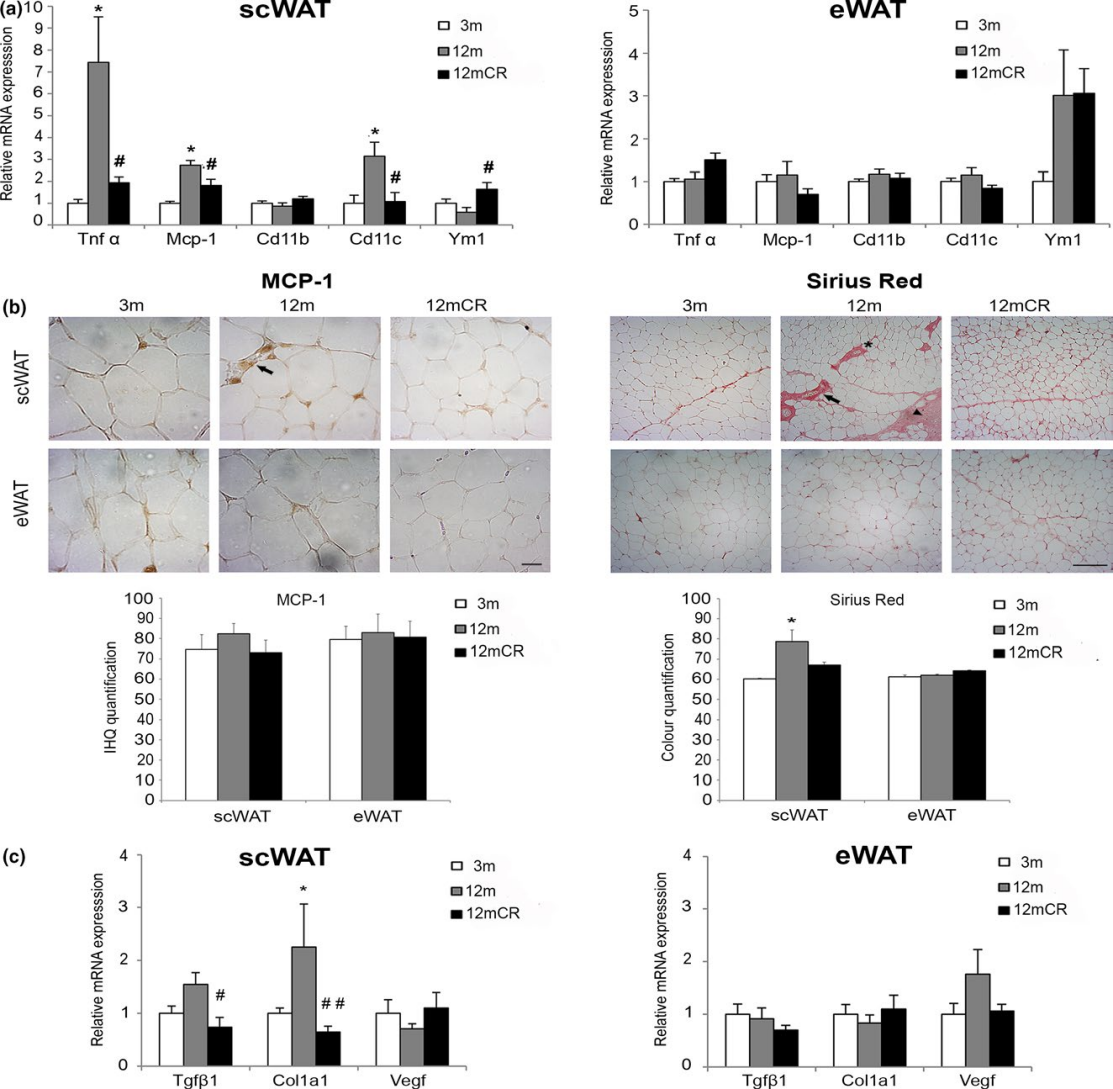
Increased fibro‐inflammation state in scWAT at early stages of aging. (a) mRNA levels of representative inflammatory genes in scWAT and eWAT in the three experimental groups. (b) Representative images of immunohistochemically analysis of MCP‐1 expression in paraffin‐embedded scWAT and eWAT from histological sections (magnification 40×, scale bar = 20μm) of the experimental groups (*n* = 4 animals/group). No immunoreaction was observed in the negative control treated with PBS without primary antibody (not shown). Arrows = locations of infiltrated MCP‐1‐expressing macrophages, which form crown‐like structures surrounding dead and dying adipocytes. Moreover, representative images of histological determination of fibrosis with Sirius red staining for collagen in paraffin‐embedded fat depots (magnification 10×, scale bar = 500μm) of the three experimental groups (*n* = 4 animals/group) are shown. Arrows = abundant fibrosis around vessels; arrowheads = collagen fibers organized in bundles containing a few adipocytes isolated from the rest of the parenchyma; asterisk = thinner collagen fibrils around adipocytes (i.e., pericellular fibrosis). (c) mRNA levels of representative fibrosis genes in scWAT and eWAT in the three experimental groups. All data are expressed as mean ± *SEM* (a, d: *n* = 7–9 animals/group). * *p* < 0.05, 12 m vs. 3 m; # *p* < 0.05, ## *p* < 0.01, 12mCR vs. 12 m

We also performed expression analyses of some inflammatory and fibrosis markers in scWAT and eWAT trying to correlate possible inflammatory signs with the impairment of scWAT to expand. Changes in the expression of several genes related to inflammation were more evident in scWAT than in eWAT. Accordingly, in scWAT, expression of the pro‐inflammatory cytokines *Tnf‐α*, *Mcp‐1, *and *Cd11c* was significantly higher in 12‐m than in 3‐m mice. Animals subjected to CR had levels of these markers like those of 3 m (Figure 3a). Moreover, the expression of *Ym1*, a macrophage anti‐inflammatory marker, was significantly higher in 12mCR animals in scWAT (Figure 3a). These differences were also observed when the levels of expression were directly compared among them in each adipose depot without referring the data to 3 m (Supporting Information Figure S3c). When differences in transcription levels were analyzed for each gene between depots, an increased pattern of expression was also observed in scWAT respect to eWAT (Supporting Information Figure S3d). Furthermore, according with the mRNA analysis pointing to an increased inflammation in scWAT from 12‐m animals, MCP‐1 staining showed the presence of the macrophage crown‐like structures in scWAT, but not in eWAT, from 12‐m mice confirming the inflammation stage (Figure 3).

Fibrosis is a tissue response to chronic inflammation. Therefore, we next analyzed the deposition of extracellular matrix in WAT depots by Sirius red staining (Figure 3b). Collagen fiber bundles were evident in scWAT, but not in eWAT, of 12‐m mice and were organized mainly in thick bands. The decreased fibrosis in scWAT from 12mCR relative to 12‐m mice detected by Sirius red staining was confirmed by gene expression of the fibrosis markers *Tgf‐β1 *and* Col1a1 *(Figure 3c).

### 2.4 Long‐term CR prevents the alterations of BAT function and thyroid axis status detected in middle‐aged mice

**Figure 4:**
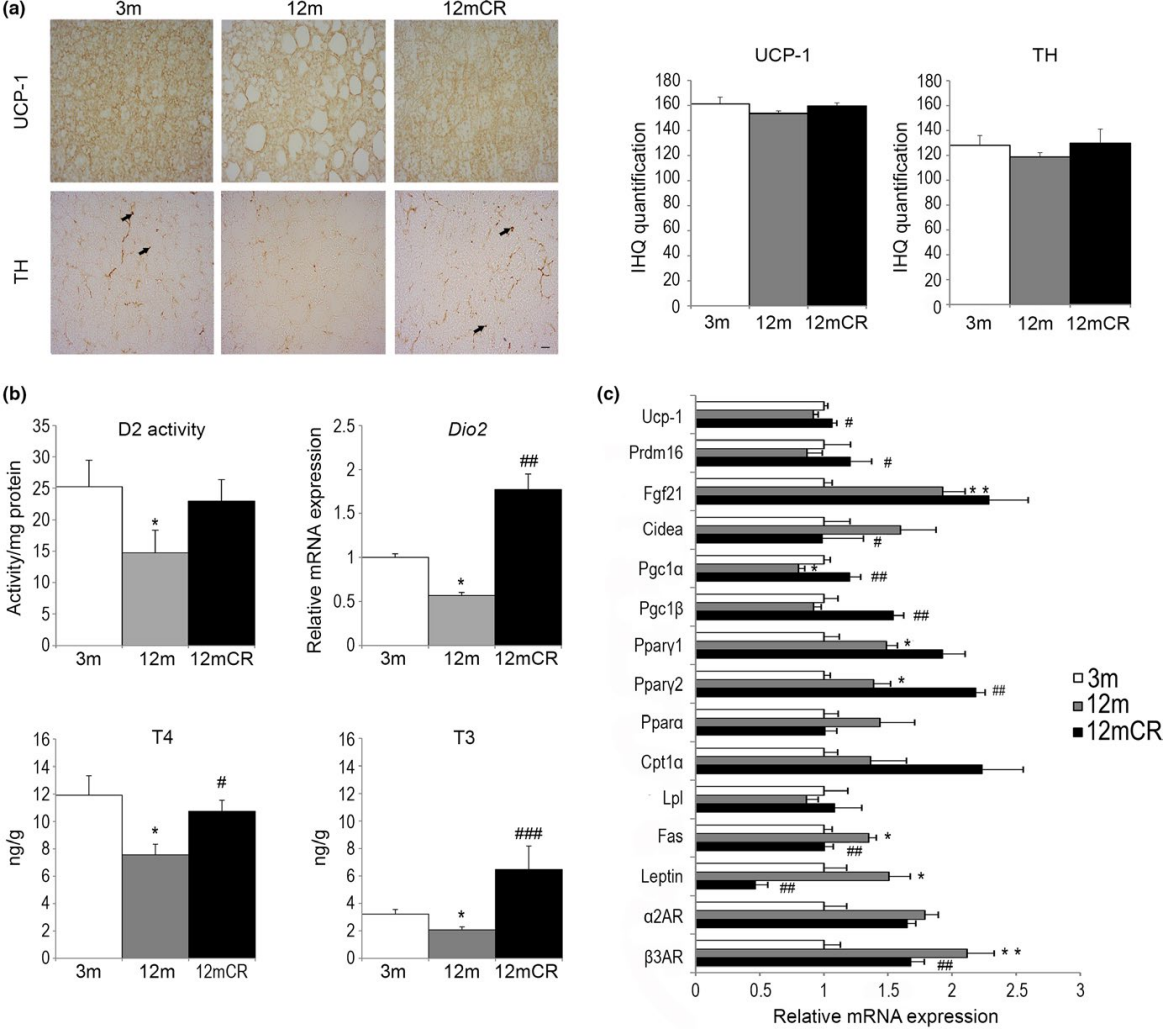
Altered BAT architecture and thyroid‐regulated energy balance are associated with initial stages of aging. (a) Representative images of immunohistochemistry for UCP‐1 and TH expression in paraffin‐embedded BAT from histological sections (magnification 40×, scale bar = 20μm) of the experimental groups (*n* = 4 animals/group). Microphotographs show changes in the size of lipid droplets in brown adipocytes within the three experimental groups; arrowheads = TH‐positive parenchymal nerve fibers in 3‐m and 12mCR mice. (b) mRNA levels of BAT activity markers in the brown fat depot in all experimental groups. (c) D2 enzymatic activity, the expression levels of *Dio2,* and T4 and T3 concentration levels in brown fat depots in the experimental groups. All data are expressed as mean ± *SEM* (a, c: *n* = 7–9 animals/group). * *p* < 0.05, 12 m vs. 3 m; # *p* < 0.05, ## *p* < 0.01, ### *p* < 0.001, 12mCR vs. 12 m

Given the differences in energy expenditure and WAT depot plasticity between 12‐m and 12mCR mice, we next explored BAT morphology. Analysis of BAT samples stained with hematoxylin and eosin (Supporting Information Figure S4) or immunostained with UCP‐1 (Figure 4a) showed that brown adipocytes were larger in 12‐m than in 3‐m mice and had a greater cytoplasmic area with the presence of larger lipid droplets, resembling WAT adipocytes. This age‐related “whitening” of BAT was not evident in CR mice, where BAT adipocytes contained multilocular lipid droplets. Moreover, although not reaching significance in density, tyrosine hydroxylase (TH) noradrenergic parenchymal fibers were better appreciated close to multilocular adipocytes in 12mCR than in 12‐m mice (Figure 4a), suggesting a possible improved response to sympathetic nervous system (SNS) activity.

Gene expression analysis of BAT showed that changes from WAT‐like morphology to BAT‐like morphology in 12mCR mice were accompanied by an increase in the expression of some BAT‐selective genes, such as *Ucp‐1*, *Prdm16, Pgc1α, *and *Pgc1β *(Figure 4c), and these data were in accordance with a tendency to increase UCP‐1 protein levels in BAT (Supporting Information Figure S4b). As previously shown during aging (Hanks et al., 2015), the mRNA level of *Fgf‐21* was higher in 12‐m and 12mCR than in 3‐m mice, and this was also observed for other adipogenic genes such as both isoforms of *Pparγ.* In agreement with the increased BAT weight (Table 1), *Fas *and *Leptin *mRNA expression was significantly higher in 12 m than in 3 m, whereas these levels were significantly lower in 12mCR mice. Moreover, *Cidea *and *β3AD *mRNA expression increased in 12‐m mice and, as anticipated, CR recovered these parameters to those of 3‐m mice (Figure 4c).

To better understand the thermogenic alterations with aging, we next measured the circulating thyroid hormones levels (Table 1). Whereas changes in thyroxine 4 (T4) levels did not reach significance among groups, triiodothyronine (T3) levels were significantly lower in 12‐m than in 3‐m mice and increased in 12mCR to the levels found in young mice.

Deiodinase 2 (D2) activity in BAT, which is related to T3 activation, was lower in 12‐m than in young animals, whereas it was higher, but not significantly, in 12mCR mice (Figure 4b). Consistent with this, the mRNA expression of *Dio2* was significantly lower in 12‐m than in 3‐m mice and was significantly higher in 12mCR animals. T4 and T3 concentrations in BAT followed a similar pattern to those of D2 activity and *Dio2* mRNA, respectively (Figure 4b), and the increase in BAT T4 suggests and increased T4 uptake in 12mCR mice according with an increased demand of T4 to produce T3. The changes in BAT T4 and T3 are parallel to those of plasma T4 and T3 (Table 1).

### 2.5 Long‐term CR improves the pattern of expression of genes related to the thermogenic response of BAT to cold exposure but fails to maintain body temperature

As a measure of BAT function, we analyzed the cold response of animals after 24 hr at 4ºC. No significant changes in BW were found after the cold challenge (data not shown) in any experimental group. Core temperature was similar in middle‐aged and young mice fed *AL*, before cold challenge; however, core temperature was lower in 12‐m compared to 3‐m mice after the cold challenge. CR decreased body temperature, which decreased even further after cold challenge (Supporting Information Figure S5).

**Figure 5:**
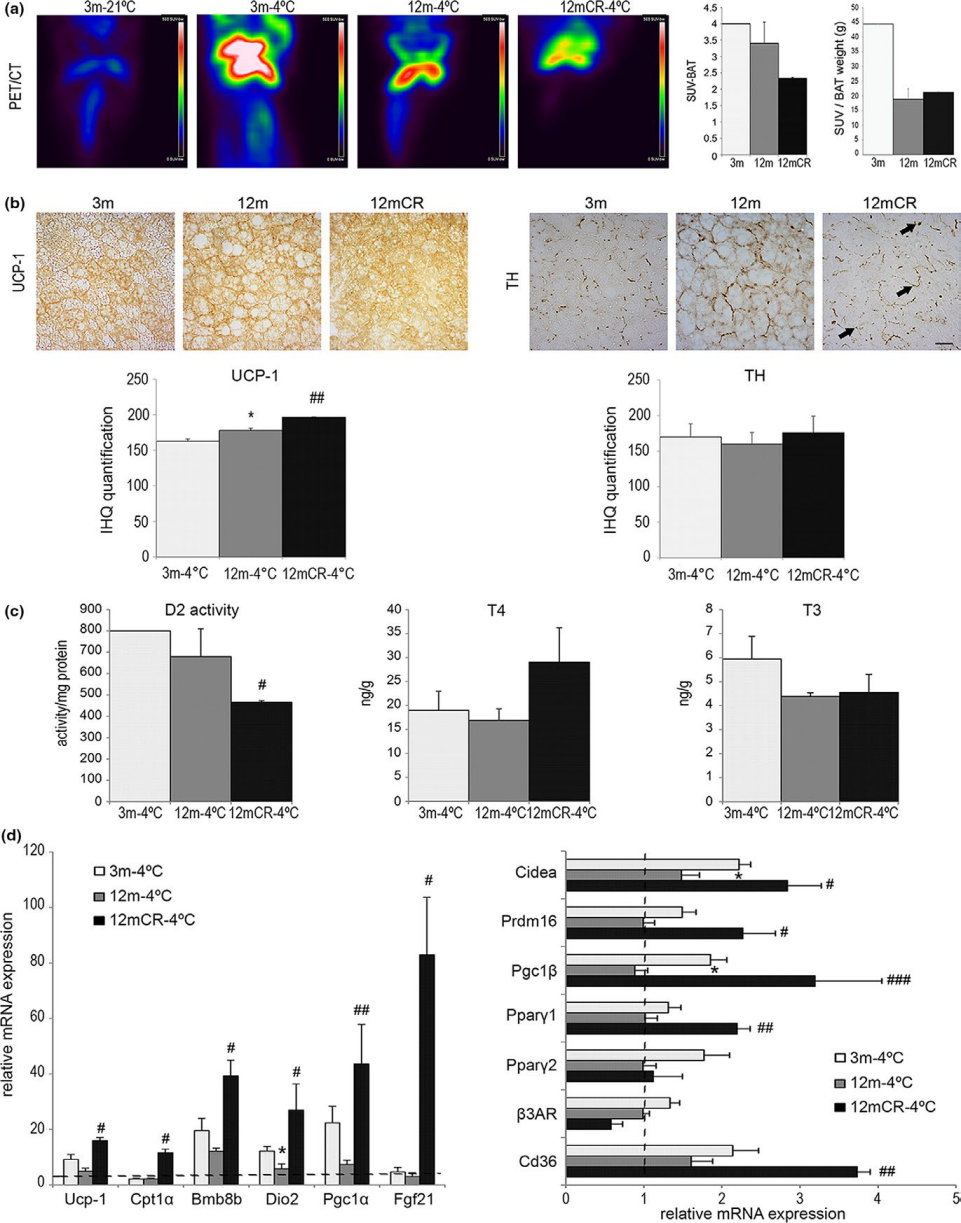
Caloric restriction improves cold exposure response in aged mice. (a) Representative fused ^18^F‐FDG PET/CT images of the experimental groups at 21 and 4ºC. Three‐month‐old mice at room temperature were used as a control for the procedure. All images show glucose uptake in the BAT, quantified as standardized uptake values (SUVs). Scale bars for ^18^F‐FDG uptake (in color) are shown on the right of the images. Areas of high ^18^F‐FDG uptake are represented in red to white colors. Data are represented as mean ± *SEM* (*n* = 2–3 animals/group). (b) Representative images of immunohistochemistry of UCP‐1 and TH localization in paraffin‐embedded BAT from histological sections (magnification 40×, scale bar = 20μm) of the experimental groups (*n* = 4 animals/group). Arrows = TH‐positive parenchymal nerve fibers in exclusively brown adipocytes. (c) D2 enzymatic activity, T4 and T3 concentration levels, in brown fat depots in the experimental groups after cold exposure. (D) mRNA levels of BAT activity markers in the three experimental groups under cold exposure compared with 3‐m mice at room temperature (dotted line). All data are expressed as mean ± *SEM* (*n* = 7–9 animals/group). * *p* < 0.05, 12 m vs. 3 m; # *p* < 0.05, ## *p* < 0.01, 12mCR vs. 12 m

We also examined ^18^F‐FDG uptake in interscapular BAT as a measurement of BAT metabolic activity (Figure 5a). In young mice, PET images revealed higher ^18^F‐FDG uptake at 4ºC than at 21ºC. However, at 4ºC, 12‐m animals showed a lower ^18^F‐FDG uptake than in 3‐m, reinforcing the idea of a decline in BAT response to cold associated with early stages of aging. CR mice showed the lowest ^18^F‐FDG SUV as compared with the other experimental groups (Figure 5a). When ^18^F‐FGD uptake was normalized by BAT weight in each group, a decrease is appreciated in 12‐m respect to 3‐m mice and CR kept this parameter low without any significant change respect to 12‐m animals (Figure 5a). Next, we analyzed BAT morphology in cold‐exposed mice by immunohistochemistry. As shown in Figure 5b, changes in UCP‐1 staining within the three experimental groups followed the pattern observed in Figure 4a. Although changes in density were not significant, localization of TH‐positive parenchymal fibers in 12mCR mice was more closely associated with adipocytes with multilocular lipid droplets than in 12‐m mice (Figure 5b).

We also evaluated the effect of cold exposure on T3 and T4 concentrations and D2 activity (Figure 5c) and mRNA expression of *Dio2* (Figure 5d) in BAT. As expected, all these values were higher under cold exposure than at 21ºC. D2 activity in BAT was significantly lower in 12mCR than in 12‐m mice, although the mRNA expression of *Dio2* was significantly higher in 12mCR (Figure 5d). This fact agrees with the requirement of insulin for the adrenergic induction of D2 activity in brown adipocytes (Martinez‐deMena & Obregón, 2005).

We detected that the mRNA levels of genes involved in BAT function were higher in animals at 4ºC than in their counterparts at 21ºC (dotted line in Figure 5d). Of note, the mRNA levels of most of the genes analyzed (*Ucp‐1*, *Cpt1α*, *Bmp8b, Dio2, Cidea, Prdm16, Pgc1α, Pgc1β, Pparγ1,* and *Fgf‐21*) were significantly higher in cold‐exposed animals under CR than in 12‐m mice, suggesting that these animals may have presented a better response of BAT. Of interest, the expression of *Cd36* (Figure 5d) was significantly higher in 12mCR mice than in the other groups, suggesting an increased utilization of lipids in the BAT of CR mice under cold exposure.

### 2.6 Cold exposure increases CR‐induced browning in scWAT in middle‐aged mice

**Figure 6:**
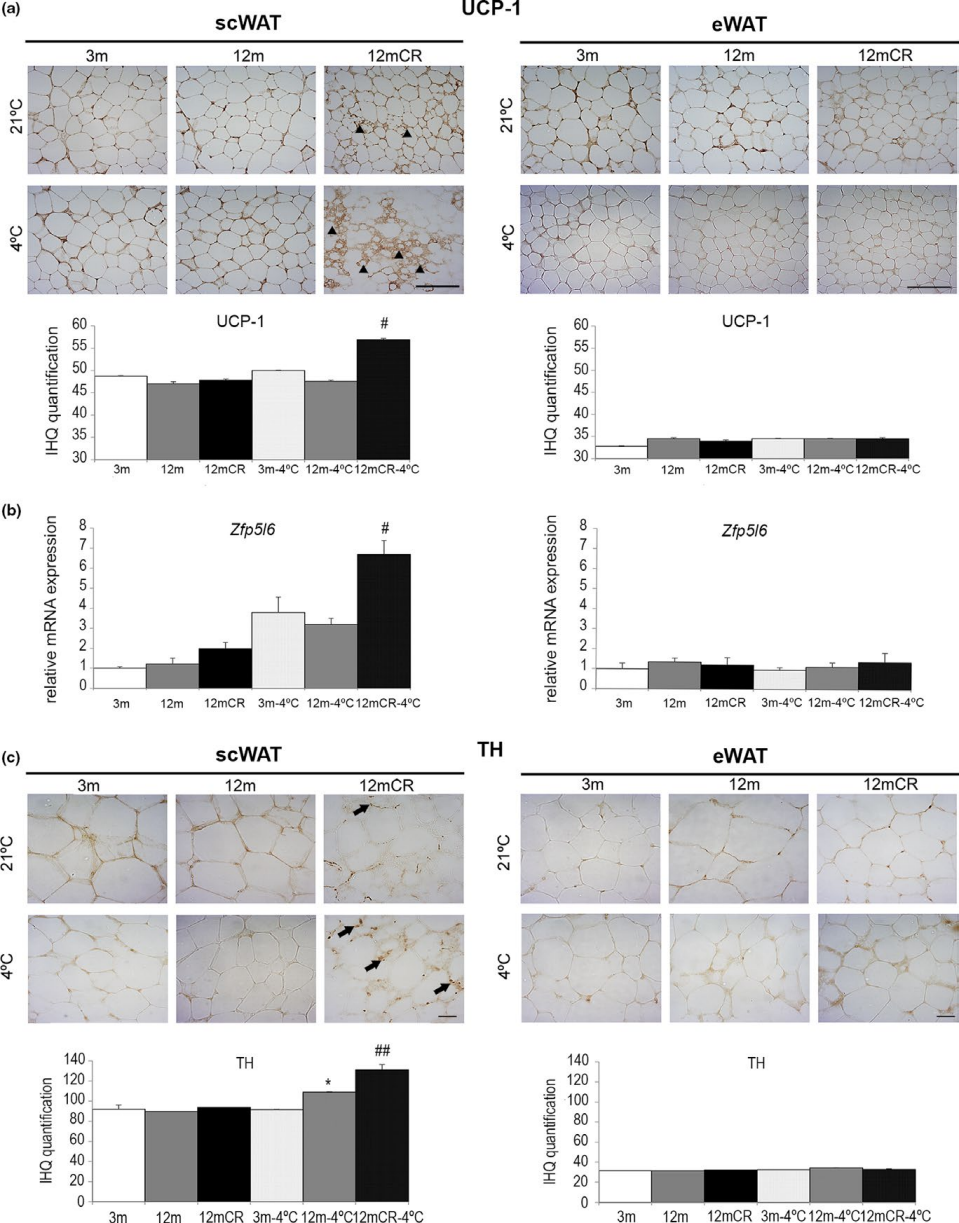
Caloric restriction‐induced browning in scWAT at early stages of aging is more pronounced after cold exposure. (a) Representative images of immunohistochemistry of UCP‐1 expression in paraffin‐embedded histological sections of scWAT and eWAT (magnification 20×, scale bar = 50μm) of the experimental groups (*n* = 4 animals/group). Arrowheads indicate adipocytes with a brown‐like phenotype (adipocytes with multilocular lipid droplets) in 12mCR mice at room temperature. A stronger effect is detected after cold exposure. (b) mRNA levels of *zfpl5* in scWAT and eWAT in all experimental groups. All data are expressed as mean ± *SEM* (*n* = 7–9 animals/group). # *p* < 0.05; 12mCR vs. 12 m. (c) Representative images of immunohistochemistry of TH expression in paraffin‐embedded histological sections of scWAT and eWAT (magnification 40×, scale bar = 20μm) of the experimental groups (*n* = 4 animals/group). Arrows = TH‐positive fibers in the parenchyma that are closely associated with adipocytes in areas containing large numbers of brown adipocytes

Aging has been described to bring about a loss of brown adipocytes in scWAT (Rogers, Landa, Park, & Smith, 2012). Thus, we explored the possible effects of aging and CR on the browning process in mice at 21 and 4ºC. Sections of scWAT and eWAT from mice were immunostained to detect UCP‐1. Although we could not appreciate significant differences on UCP‐1 immunostaining between young and middle‐aged mice, a strongest immunoreactivity for UCP‐1 in scWAT was found in 12mCR animals at 4ºC (Figure 6), indicating an induced beige‐like appearance. This was confirmed by increased gene expression of *zfp5l6*, a browning marker, in 12mCR compared to 12‐m mice at 4ºC. Noradrenergic parenchymal fibers were greater in 12mCR mice at 4ºC determined by TH staining. These effects were not observed in eWAT (Figure 6).

## 3 DISCUSSION

Aging has been associated with the development of peripheral IR and other metabolic complications. CR ameliorates peripheral IR during aging identifying adiposity as a potential key factor in the development of age‐associated IR. Here, our data demonstrate that middle‐aged (12 m) SV129 mice already show peripheral IR, as deduced by HOMA_IR_ and ITT data. Older animals (20 m) show more difficulties to maintain glucose homeostasis, as deduced by the significant increased fasting glycemia, HOMA_IR_, and the deterioration of pancreatic islets, confirming the progressiveness of this age‐associated alteration. CR significantly improved insulin global sensitivity in middle‐aged animals. Although CR also seemed to ameliorate IR in older animals, this was achieved to a lesser extent, as deduced by the lack of significance in the recovery of several parameters such as the decrease of fasting insulin, the increase of the slope in the ITT, and the HOMA_IR_ data, in spite of a longer period of CR (7 months in 12‐m mice vs. 17 months in 20‐m mice). These findings are in line with previous data in which older animals showed a poorer recovery of IR after three months of CR (Escriva et al., 2007). Therefore, aside from possible differential effects of CR depending on the age and, the metabolic status of the animals, an early intervention as described herein, which allows a longer time of food restriction, seems to be a better option to prevent age‐associated metabolic alterations. Besides IR, middle‐aged SV129 mice also showed several alterations in white and brown adipose depots, confirming that at this age several symptoms of the aging process were already evident. Our data point to altered WAT and BAT plasticity as well as alteration of thyroid axis status, as important factors in the development of systemic and local IR in middle‐aged mice.

WAT is a critical regulator of systemic energy homeostasis, and its remodeling under pathophysiological and challenging conditions is linked to metabolic complications such as IR (Huh, Park, Ham, & Kim, 2014). AT plasticity in obesity, as well as in aging, involves tissue expandability (hypertrophy and/or hyperplasia), recruitment of inflammatory cells, and vascularization and innervation (Carobbio et al., 2017; Wang et al., 2014). Accretion of visceral, rather than scWAT, has been associated with the development of IR and metabolic syndrome; however, scWAT also must undergo expansion to accommodate an increased lipid supply to avoid ectopic lipid accumulation causing lipotoxicity. In our study, although the weight of both eWAT and scWAT was greater in middle‐aged animals, only eWAT showed increased adipocyte size with aging, suggesting that hyperplasia rather than hypertrophy is responsible for the increase in the weight of scWAT at middle age. On the other hand, a limited capability of adipocytes from scWAT to keep on with the expansion may be related to the increased inflammation and fibrosis detected in this fat depot at this age, as established by the increased expression of inflammatory and fibrosis markers. Studies in vitro have shown that preadipocytes in contact with inflammatory cells, such as macrophages, can be high‐level “producers” of selective fibrotic molecules including collagen (Sun, Tordjman, Clément, & Scherer, 2013). Our results show that scWAT, but not eWAT, developed inflammation and fibrosis at 12 m. This inflammatory and fibrotic process in scWAT may be related to early metabolic complications such as the systemic IR already present in middle‐aged animals as demonstrated herein. Nevertheless, because of its greater overall weight, eWAT may gain relevance in the development of global inflammation and other metabolic complications in more advanced stages of aging and obesity. This would also fit with the higher and sustained enlargement of eWAT adipocytes as compared with scWAT along the aging process. In addition to the altered adipocyte expandability, scWAT showed more metabolic alterations than eWAT at middle age, as deduced from the decreased expression of genes involved in lipid metabolism, adipogenesis, and oxidative capacity. CR clearly reverses, at least in part, the age‐related alterations in the expression of many of these genes, pointing to a beneficial role of intervention. The fact that CR affects the basal expression of genes involved in both catabolism and anabolism could be explained as an improvement of the plasticity of the tissue in order to respond to different metabolic circumstances.

As previously mentioned, BAT has been postulated as a potential target for treatment against metabolic disorders (Arita et al., 1999; Cypess et al., 2009; van der Lans et al., 2013). Along this line, a decline of BAT function has been associated with aging in humans (Saito et al., 2009; Yoneshiro et al., 2011), and in rodents (Sierra Rojas et al., 2016). Our data show that middle‐aged mice already manifest structural alterations with increased unilocular droplets resembling those of white adipocytes, what suggests a “whitening” or loss of function of BAT. Age‐induced BAT alterations also affect thyroid functionality with likely metabolic consequences. An examination of the expression of BAT genes with an important role in mitochondrial biogenesis or regulated by the thyroid axis agrees with the apparent loss of BAT function in middle‐aged animals. The increased expression of *β3AR* may also reflect a mechanism to compensate for the lower adrenergic stimulation shown by TH staining, as previously suggested (Rogers et al., 2012).

CR was accompanied by a significant higher expression of thermogenic genes analyzed as well as several genes involved in BAT differentiation. This, together with the recovery of a more brown‐like morphology, showing fewer unilocular adipocytes, suggested an improvement of BAT function or capability to respond to metabolic challenges. This also would fit with the improvement of the thyroid axis status induced by CR, as deduced from thyroid hormone levels, D2 activity, and the expression of several genes related to BAT function.

We also studied the physiological response of BAT to cold exposure analyzing glucose uptake by PET. A lower ^18^F‐FDG uptake in response to cold in middle‐aged animals was found, supporting the above‐mentioned decline in BAT function already detected in 12‐m mice. Impaired BAT function may well contribute to the development of IR during the aging process. Contrary to what it might have been expected, CR did not recovered ^18^F‐FDG uptake even when this parameter was normalized by BAT weight, suggesting a limited capability of BAT to metabolize glucose in these conditions. Moreover, core body temperature, energy expenditure, and locomotor activity were reduced in CR mice compared to *AL *mice which is in agreement with previous studies (Golightly, Boys, Cameron, & Zglinicki, 2012). Reduction in body temperature has been described consistently in the literature as one of the consequences of CR in several animal models, including humans (Barger et al., 2017; Duffy et al., 1989; Mattison et al., 2017; Mitchell et al., 2016; Soare, Cangemi, Omodei, Holloszy, & Fontana, 2011). This has been proposed as a measure to preserve energy under CR to ensure a better survival. This would be in agreement with the lower locomotor activity and the decreased energy expenditure observed in our CR animals. In line with this, inverse relationship between body temperature and longevity has been postulated (Keil, Cummings, & Magalhães, 2015) to operate in several ways such as suppression of autoimmunity (Rikke & Johnson, 2004) or decreased ROS production (Carrillo & Flouris, 2011). The increased induction of several thermogenic genes, the recovered morphology, and the improvement of the thyroid hormone status in BAT suggests that long‐term CR might improve BAT response capability and function. The lack of recovery in ^18^F‐FDG uptake in 12mCR at cold exposure may be explained, at least in part, by the increased expression of *Cd36 *in these animals, which is involved in lipid uptake. This would lead to a shift toward lipid consumption in BAT as consequence of carbohydrates scarcity under CR. This also would be in agreement with the rapid decreased RER in the second phase of the dark cycle, in CR mice when these animals leave to behave like ad libitum, probably because as soon as they finish the available food (carbohydrates), they start using fat faster than *AL* animals, demonstrating metabolic flexibility in this phase.

It is worth noting the effect of aging and CR on scWAT browning. In parallel to a possible improved BAT function in CR animals, we observed the emergence of beige adipocytes with increased UCP‐1 and TH expression in scWAT, but not in eWAT. This was more evident in cold‐induced thermogenesis, even without cold acclimation as previously described (Murano, Barbatelli, Giordano, & Cinti, 2009). These data seem to contrast with others (Rogers et al., 2012), which described that after 10 months of 40% CR, no BAT‐like areas were detected in scWAT from 12‐m animals. Nevertheless, in line with our data, the presence of multilocular cells and an attenuation of the age‐associated fall in the expression of genes involved in the thermogenic response such as *Cidea*, *Cox7a1,* and *Cox8b* were also described in scWAT from 12mCR animals in the same article. We speculate that increased thyroid function induced by CR and the stimulated β‐adrenergic pathway during cold‐induced thermogenesis could induce BAT to significantly upregulate genes and secrete molecules to promote browning of scWAT in CR middle‐aged mice. Nevertheless, the scarcity of fuel and energy reservoir may limit some of the ameliorating effects of CR on aging metabolism and, particularly, the thermogenic response of BAT under a long‐term cold exposure. The fact that CR has a strong effect on adiposity while improves several metabolic alterations, points to adiposity as one of the causes of the deleterious effects of aging. This also agrees well with the deleterious effects on metabolic parameters and lifespan that increased adiposity, by means of genetic alterations and/or by high‐fat diets, induces in many animal models. However, in spite of the improvement, CR does not fully reverse metabolic alterations suggesting that metabolic alterations associated with aging cannot be solely explained in terms of adiposity. Further work with adiposity‐matched animals would be necessary to clarify the precise role of adiposity in the aging process.

In conclusion, our results support the notion that limited scWAT expandability and decreased activity of thyroid hormones in BAT may also be related to the development of IR at middle age. Moreover, long‐term CR improves many of the underlying metabolic alterations associated with early signs of aging such as IR, loss of scWAT plasticity, impaired thyroid axis status, and BAT functionality. Nevertheless, scarcity of fuel and energy reservoir may limit some of the ameliorating effects of CR on aging metabolism under long‐term challenges such as cold.

## 4 EXPERIMENTAL PROCEDURES

### 4.1 Animals and diets

All animal procedures conformed to European Union laws and guidelines for animal care, and experimental procedures were approved by the ethics committee of the Universidad Rey Juan Carlos (Spain). Male 129S2/SvPasCrl mice (Charles River Laboratories) were used for all studies and were housed individually in climate‐controlled quarters (21ºC) with a 12‐hr light/dark cycle. Food (13% calories derived from fat; Research diet#2014C, Envigo) and water were available ad libitum unless otherwise stated. In some experiments, mice were randomly assigned to undergo 20% CR from 3 months of age, as described earlier (Sierra Rojas et al., 2016; Speakman & Mitchell, 2011). The restricted food was administrated at 19 hr just before the dark period starts (from 20 to 8 hr). For cold exposure, animals were housed in their individual cages in a cold chamber (4ºC) for 24 hr. Animals were sacrificed by cervical dislocation at fed state, and selected tissues were kept for analysis.

### 4.2 Glucose and insulin tolerance testing

Glucose tolerance test (GTT) and insulin tolerance test (ITT) were performed as described (Vivas et al., 2016). The ITT slopes from 0 to 10 min were calculated as Δ(glucose 10 min–glucose 0 min)/10 min.

### 4.3 Indirect calorimetry and activity measurements

Animals were placed in a comprehensive laboratory animal monitoring system (PhenoMaster, TSE Systems, Germany). Energy expenditure, the respiratory exchange rate (RER), and locomotor activity were calculated according to the protocols provided by the manufacturer (http://www.tse-systems.com).

### 4.4 Biochemical analyses and deiodinase activity in plasma and BAT

Serum insulin levels were measured by ELISA kit (Mercodia AB) following the user's manufacturer. Serum adipokines and cytokines were determined with the Bio‐Plex platform (Bio‐Plex Pro^TM^ Diabetes Assays, Bio‐Rad (http://www.bioplex)). The estimate of IR by HOMA_IR_ score was calculated with the following formula: fasting serum insulin (µU/ml) × fasting plasma glucose (mmol/L)/22.5 as described (Galvin et al., 1992).

Triiodothyronine (T3) and thyroxine (T4) were quantified by specific and highly sensitive radioimmunoassay in plasma and BAT, after appropriate extraction and purification of tissue extracts as described (Morreale De Escobar, Pastor, Obregon, & DelRey, 1985). High specific activity T4 and T3 labeled with ^125^I were synthesized as described (Obregon, Morreale de Escobar, & Escobar del Rey, 1978). Type II 5´ iodothyronine deiodinase (D2) activity was assayed as previously described (Obregón et al., 1989) in homogenates (1:30, weight/vol) using ^125^I‐T4, 2 nM T4, 1 µM T3, and 20 mM dithiothreitol (DTT) and 1 mM propylthiouracil for 1 hr at 37º C. Results are expressed in femtomoles *per* hour *per* milligram of protein.

### 4.5 Positron emission tomographic‐computed tomographic (PET/CT) imaging

Mice were in vivo imaged with the Inveon MM small animal SPECT/PET/CT scanner (Siemens Medical Solutions, Knoxville, TN, USA). Thirty‐minute‐long PET scans were acquired 60 min after injection of ^18^F‐FDG (7.4 MBq). Mice were maintained under isoflurane anesthesia during injection, uptake, and imaging. All animals had a CT scan before the PET scan for attenuation correction and anatomical delineation of PET images. All PET images were calibrated in Bq/cm^3 ^and converted to standard uptake value (SUV) units (Auffret et al., 2012). All in vivo images were analyzed using Inveon Research Workplace software (Siemens Medical Solutions, USA).

### 4.6 Histology, immunohistochemistry, and western blotting analysis

Bouin‐fixed samples were gradually dehydrated and embedded in paraffin, cut to 4 µm, and stained with Harris hematoxylin (Sigma Co., St. Louis, USA) or incubated with primary antibody UCP‐1 (Abcam), MCP‐1 (Santa Cruz Biotechnology, Inc.), insulin (Thermo Fisher), and TH (Millipore) for immunohistochemistry as previously reported (Martínez‐García et al., 2015). Morphological examination of fibrosis was performed using Sirius red staining for collagen.

To measure the area of adipocytes, four nonconsecutive sections per animal were quantified (*n* = 4 animals) using ImageJ 1.45 (National Institutes of Health, Bethesda, MD, USA) and Adiposoft software (http://fiji.sc/Adiposoft).

Protein lysates from mouse tissues were electrophoresed in SDS‐PAGE gels (10%), and Western blotting was performed as described before (López et al., 2010; Martínez‐García et al., 2015) with the following antibodies: PPARγ1 (Santa Cruz Biotechnology, Inc.), PPARγ2 (Santa Cruz Biotechnology, Inc.), ATGL (Cell Signaling Technology), UCP‐1 (Abcam), SIRT1 (Santa Cruz Biotechnology, Inc.) β‐actin (Sigma‐Aldrich). Quantification was performed with the ImageJ 1.45 software (National Institutes of Health, Bethesda, MD, USA). Values were expressed relative to β‐actin levels.

### 4.7 RNA isolation and gene expression analysis

RNA extraction and quantitative RT–PCR were performed as reported (Martínez‐García et al., 2015; Vivas et al., 2016). The input value of the gene of interest was normalized against β2‐microglobulin or β‐actin as internal controls. Real‐time RT–PCR was performed using the specific primers described in the Supplementary data. The 2^‐ΔΔCt^ method was employed to calculate the relative index of gene expression.

### 4.8 Statistical analysis

All data are presented as mean ± *SEM*. Statistical analysis was carried out with GraphPad Prism software (San Diego, CA, USA). Results were analyzed with a Shapiro–Wilk normality test. After that, data were analyzed with a Kruskal–Wallis test to check the statistical differences among experimental groups. Finally, experimental groups were compared by Mann–Whitney's nonparametric test. A *p* value < 0.05 was considered significant.

## CONFLICT OF INTEREST

None declared.

## AUTHOR CONTRIBUTION

P.C, Y.V., M.J.O., and G.M.G. conceived the study, researched data, and wrote the manuscript, which was then read and approved by all the co‐authors (A.I.L., D.H., P.S.C., I.V., L.T., Y.L., C.M.). M.R., M.J.O., and M.L. contributed to discussion, and reviewed and edited the manuscript. A.I.L. and C.M. reviewed the manuscript. P.C., M.R., and G.M.G. are the guarantors of this work. The authors declare that there is no duality of interest associated with this manuscript.
