# Supplementary material for: Long‐term caloric restriction ameliorates deleterious effects of aging on white and brown adipose tissue plasticity
Source: Aging Cell. 2019 Mar 28;18(3):e12948. doi: 10.1111/acel.12948 (PMC6516146; doi:10.1111/acel.12948)
Supplement: Supplementary file 1 [file ACEL-18-e12948-s001.docx]

**Table S1. Oligonucleotide primers for qPCR.**

| 18s | Forward | CGGCTACCACATCCAAGGAA |
| --- | --- | --- |
|  | Reverse | GTCGGAATTACCGCGGCT |
|  | Probe | GAGGGCAAGTCTGGTGCCAG |
| Adiponectin | Forward | CAGTGGATCTGACGACACCAA |
|  | Reverse | TGGGCAGGATTAAGAGGAACA |
|  | Probe | AGGGCTCAGGATGCTACTGTGCAAGC |
| α2AR | Forward | CGCAGGCCATCGAGTACAA |
|  | Reverse | GGTGACAATGATGGCCTTGAT |
| Atgl | Forward | CGCCTCTCGAAGGCTCTCT |
|  | Reverse | TGTAGCCCTGTTTGCACATCTC |
| β3AR | Forward | CCAGCCAGCCCTGTTGA |
|  | Reverse | GGACGCGCACCTTCATAGC |
| β2mg | Forward | ACTGATACATACGCCTGCAGAGTT |
|  | Reverse | TCACATGTCTCGATCCCAGTAGA |
| βactin | Forward | GCTCTGGCTCCTAGCACCAT |
|  | Reverse | GCCACCGATCCACACAGAGT |
| Bmp8b | Forward | CATGACGGATGACAGTGGCG |
|  | Reverse | AGCAGGGATCTGGGTTAGGT |
| Cd11b | Forward | TGCCATAATGCAAGTTGCTG |
|  | Reverse | ATCACCAGCAAAGTGGAAGC |
| Cd11c | Forward | CAACCCATGCTGCTGGCTGTAGA |
|  | Reverse | TAAGGTCATCCTGGCAGATGTG |
| Cd36 | Forward | GCCAAGCTATTGCGACATGA |
|  | Reverse | TCTCAATGTCCGAGACTTTTCAAC |
|  | Probe | CACAGACGCAGCCTCCTTTCCACCT |
| Cidea | Forward | GTGGACACAGAGGAGTTCTTT |
|  | Reverse | GTCGAAGGTGACTCTGGCTATTC |
|  | Probe | ACAGAAATGGACACCGGG |
| Col1α1 | Forward | CCTGGCAAAGACGGACTCAAC |
|  | Reverse | GCTGAAGTCATAACCGCCACTG |
| Cpt1α | Forward | CCTGGGCATGATTGCAAAG |
|  | Reverse | GCCACTCACGATGTTCTTCGT |
| Cpt1β | Forward | GCGTGCCAGCCACAATTC |
|  | Reverse | TCCATGCGGTAATATGCTTCAT |
|  | Probe | CCGGTSCTTGGSTTCTGTGCGGCC |
| Cox7a1 | Forward | GGGAATGGACAACGTCCTGT |
|  | Reverse | GCCCAGCCCAAGCAGTATAA |
| Cox8b | Forward | CCAGCCAAAACTCCCACTTC |
|  | Reverse | GGCTAAGACCCATCCTGCTG |
| Cpt1α | Forward | CCTGGGCATGATTGCAAAG |
|  | Reverse | GCCACTCACGATGTTCTTCGT |
| Dgat2 | Forward | GCTGGCATTTGACTGGAACA |
|  | Reverse | GCCACACGGCCCAGTTT |
| Fas | Forward | GCCCAGACAGAGAAGAGGCA |
|  | Reverse | CTGACTCGGGCAACTTCCC |
| Fgf21 | Forward | ATGGTTCTCCGTCGAAGGACT |
|  | Reverse | GAGGCTTCAAGCTCACAGGG |
| Hsl | Forward | GGAGCACTACAAACGCAACGA |
|  | Reverse | TCGGCCACCGGTAAAGAG |
| Leptin | Forward | CCAGGATGACACCAAAACCCT |
|  | Reverse | GCTGGTGAGGACCTGTTGAT |
| Lpl | Forward | TGGAGAAGCCATCCGTGTG |
|  | Reverse | TCATGCGAGCACTTCACCAG |
| Mcp-1 | Forward | GGCTCAGCCAGATGCAGTTAA |
|  | Reverse | CCTACTCATTGGGATCATCTTGC |
| Mfn2 | Forward | AGGTCAGGGGTATCAGCGAA |
|  | Reverse | GATCACGGTGCTCTTCCCAT |
| mt12S | Forward | TTGGTAAATTTCGTGGCAGCCACC |
|  | Reverse | CAGTTTGGGTCTTAGCTGTCGTGT |
| mtAtp6 | Forward | CAGTCCCCTCCCTAGGACTT |
|  | Reverse | TCAGAGCATTGGCCATAGAA |
| mtCo1 | Forward | CTCGCCTAATTTATTCCACTTCA |
|  | Reverse | GGGGCTAGGGGTAGGGTTAT |
| mtCo2 | Forward | ACCTGGTGAACTACGACTGCTAGA |
|  | Reverse | TGCTTGATTTAGTCGGCCTGGGAT |
| mtCytB | Forward | ACCAATCTCCCAAACCATCA |
|  | Reverse | TCCAGAGACTTGGGGATCTAAC |
| mtND1 | Forward | GGGATAACAGCGCAATCCTA |
|  | Reverse | ATCGTTGAACAAACGAACCA |
| mtTFAM | Forward | CAGGAGGCAAAGGATGATTC |
|  | Reverse | CCAAGACTTCATTTCATTGTCG |
| Nox2 | Forward | AGTGCGTGTTGCTCGACAAG |
|  | Reverse | CCAAGCTACCATCTTATGGAAAGT |
|  | Probe | CAACTGGACAGGAACCT |
| Pgc1α | Forward | AACCACACCCACAGGATCAGA |
|  | Reverse | CTCTTCGCTTTATTGCTCCATGA |
|  | Probe | CAAACCCTGCCATTGTTAAGACCGAGAA |
| Pgc1β | Forward | GGCCTTGTGTCAAGGTGG AT |
|  | Reverse | GGTGCTTATGCAGTTCCGTAC A |
|  | Probe | AGACCCCCACACTGCGGGCTC |
| Pparα | Forward | CCTCAGGGTACCACTAGGGAGT |
|  | Reverse | GCCCGAATAGTTCGCCGAAA |
| Pparγ1 | Forward | TTTAAAAACAAGACTACCCTTTACTGAAATT |
|  | Reverse | AGAGGTCCACAGAGCTGATTCC |
|  | Probe | AGAGATGCCATTCTGGCCCCACCAACTT |
| Pparγ2 | Forward | GATGCACTGCCTATGAGCACTT |
|  | Reverse | AGAGGTCCACAGAGCTGATTCC |
|  | Probe | AGAGATGCCATTCTGGCCCCACCAACTT |
| Prdm16 | Forward | CCCAGGAGAGCTGCATCAAA |
|  | Reverse | AGGAACTCGCTACACGGATG |
| Scd1 | Forward | CTTGCGGATCTTCCTTATCATT |
|  | Reverse | GATCTCGGGCCCATTCG |
|  | Probe | ACCATGGCGTTCCAGAATGACGTGT |
| Tgfβ1 | Forward | TGAACCAAGGAGACGGAATACA |
|  | Reverse | CACGTGGAGTTTGTTATCTTTGCT |
| Tnfα | Forward | CCAGACCCTCACACTCAGATC |
|  | Reverse | CACTTGGTGGTTTGCTACGAC |
| Ucp-1 | Forward | CCCGCTGGACACTGCC |
|  | Reverse | ACCTAATGGTACTGGAAGCCTGG |
|  | Probe | AAGTCCGCCTTCAGATCCAAGGTGAAG |
| Vegf | Forward | GTGCACTGGACCCTGGCTTTACTG |
|  | Reverse | ATGTGCTGGCTTTGGTGAGGTTT |
| Ym1 | Forward | ACTTTGATGGCCTCAACCTG |
|  | Reverse | AATGATTCCTGCTCCTGTGG |


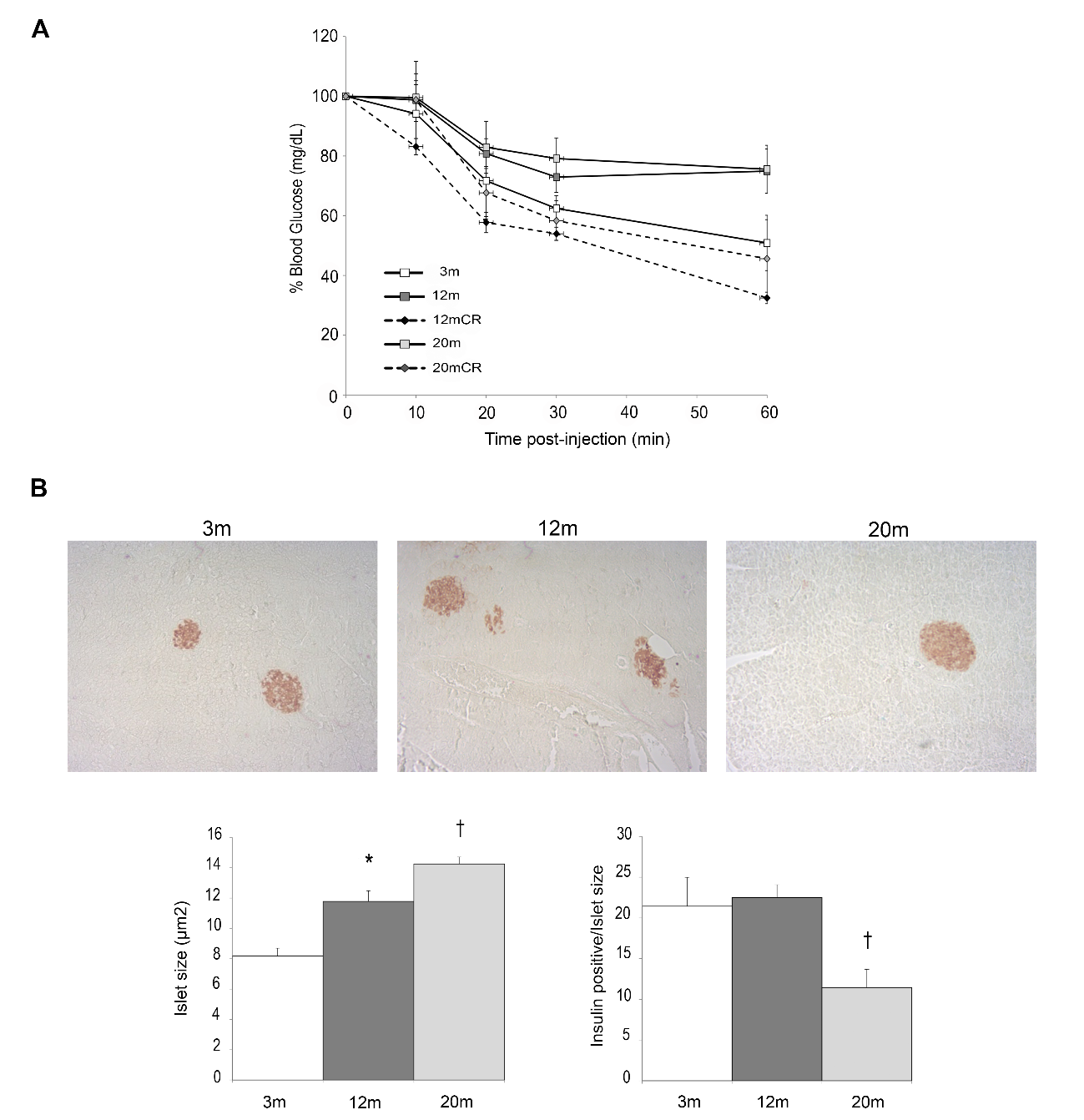


**Figure S1. ITT in % glucose and progressive pancreatic effects with aging.** (A) ITT in % of basal glucose curves from 3m, 12m, 12mCR, 20m and 20mCR mice. (B) Insulin staining of pancreatic sections (magnification 10×, scale bar = 500μm). Islet area (µm^2^) and insulin positive staining relative to the total islet area. (B) AUC of the ITT from 3m, 12m, 12mCR, 20m and 20mCR mice. Data are expressed as mean ± SEM (A: *n* = 7-9 animals/group; B: *n* = 4-5 animals/group). ^*^*P*<0.05; †*P*<0.05, 20m *vs.* 12m.


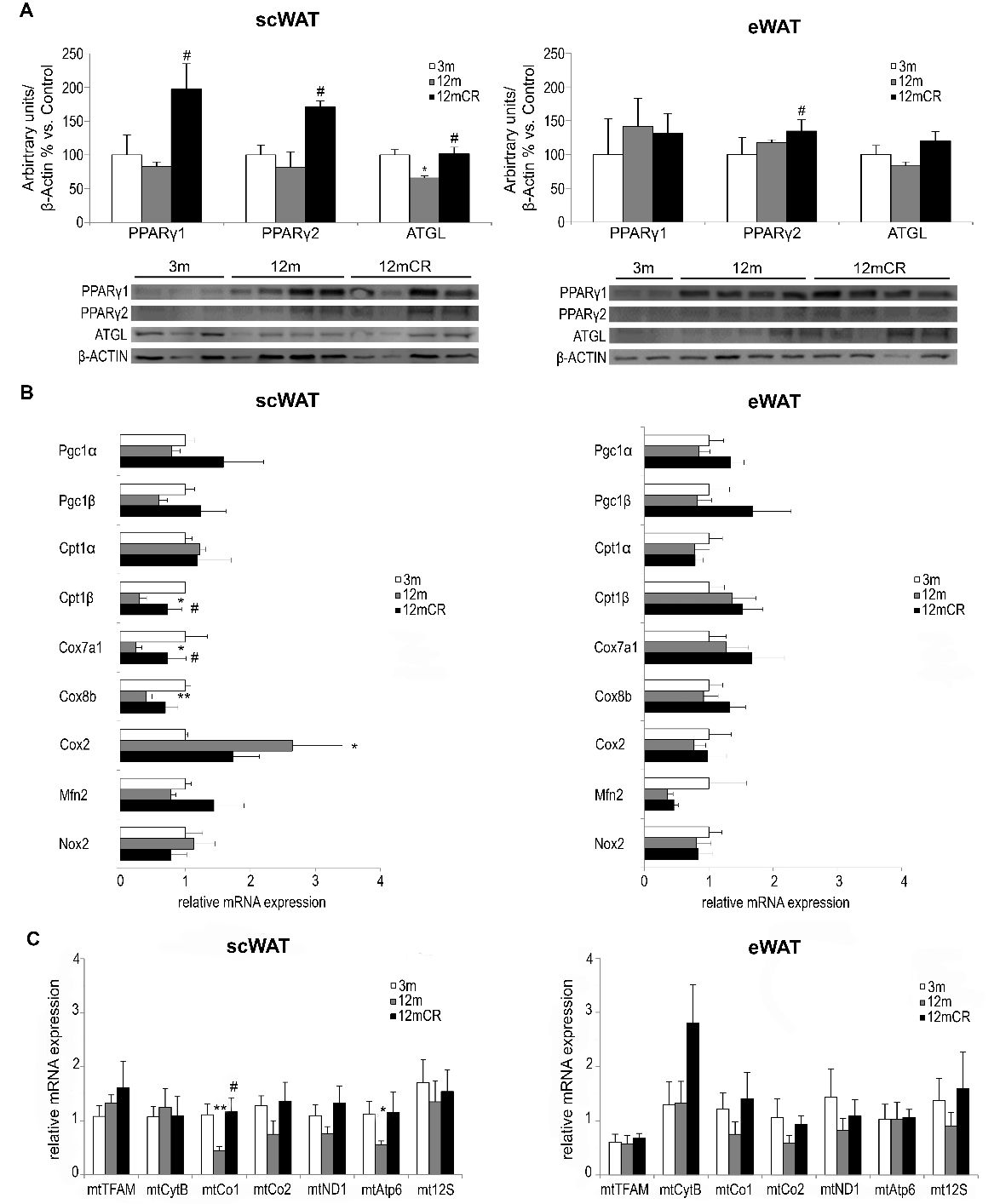


**Figure S2. Lipogenic protein measurements, mRNA expression of genes involved in mitochondrial biogenesis and oxidative capacity** **in scWAT and eWAT**. (A) Quantification of PPARγ1, PPARγ2 and ATGL total protein expression in scWAT and eWAT. Levels of protein were normalized to total β-actin. All data are expressed as mean ± SEM (*n* = 4–5 animals/group). (B) mRNA levels of representative oxidative genes in scWAT and eWAT in the three experimental groups. (C) mRNA levels of representative mitochondrial genes in scWAT and eWAT in the three experimental groups. All data are expressed as mean ± SEM (*n* = 7–9 animals/group). ^*^*P* < 0.05, 12m *vs.* 3m; ^**^*P* < 0.05, 12m *vs.* 3m; ^#^*P* < 0.05, 12mCR *vs.* 12m; ^##^*P* < 0.01, 12mCR *vs.* 12m.


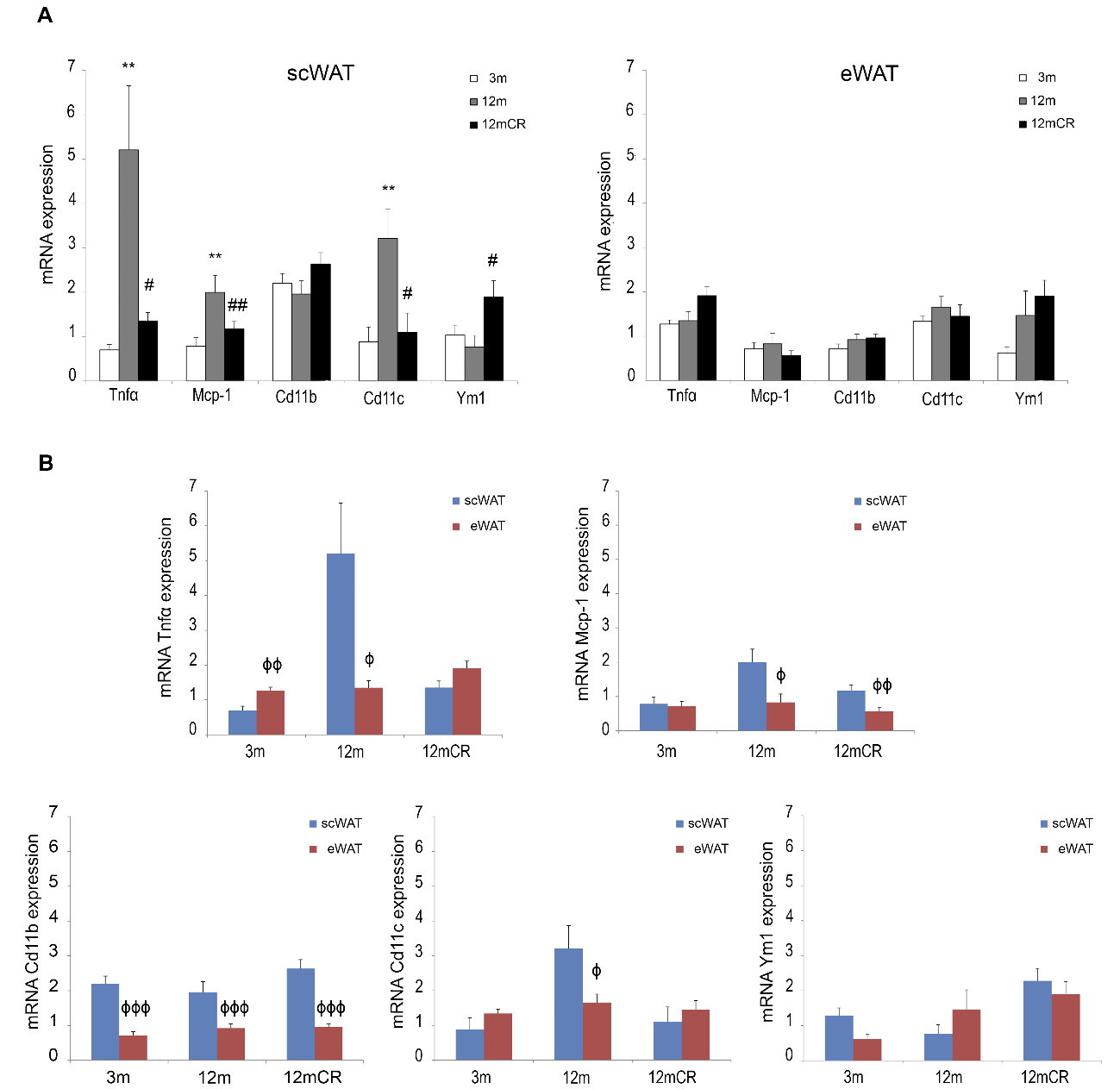


**Figure S3. Differences in inflammatory and fibrosis transcript levels in scWAT and eWAT.** Differences in inflammatory and fibrosis transcript levels in scWAT and eWAT. (A) Differences in inflammatory and fibrosis transcripts levels among genes between 3m, 12m and 12mCR mice without relativizing to 3m. (B) Differences for each inflammatory and fibrosis gene between scWAT and eWAT. Data are expressed as mean ± SEM (*n* = 7–9 animals/group). ^**^*P*<0.01 12m *vs.* 3 m; ^#^*P*<0.05; ^##^*P*<0.01 12mCR *vs.*12m; ^ϕ^*P*<0.05; ^ϕϕ^*P*<0.01; ^ϕϕϕ^*P*<0.001 scWAT *vs.* eWAT.


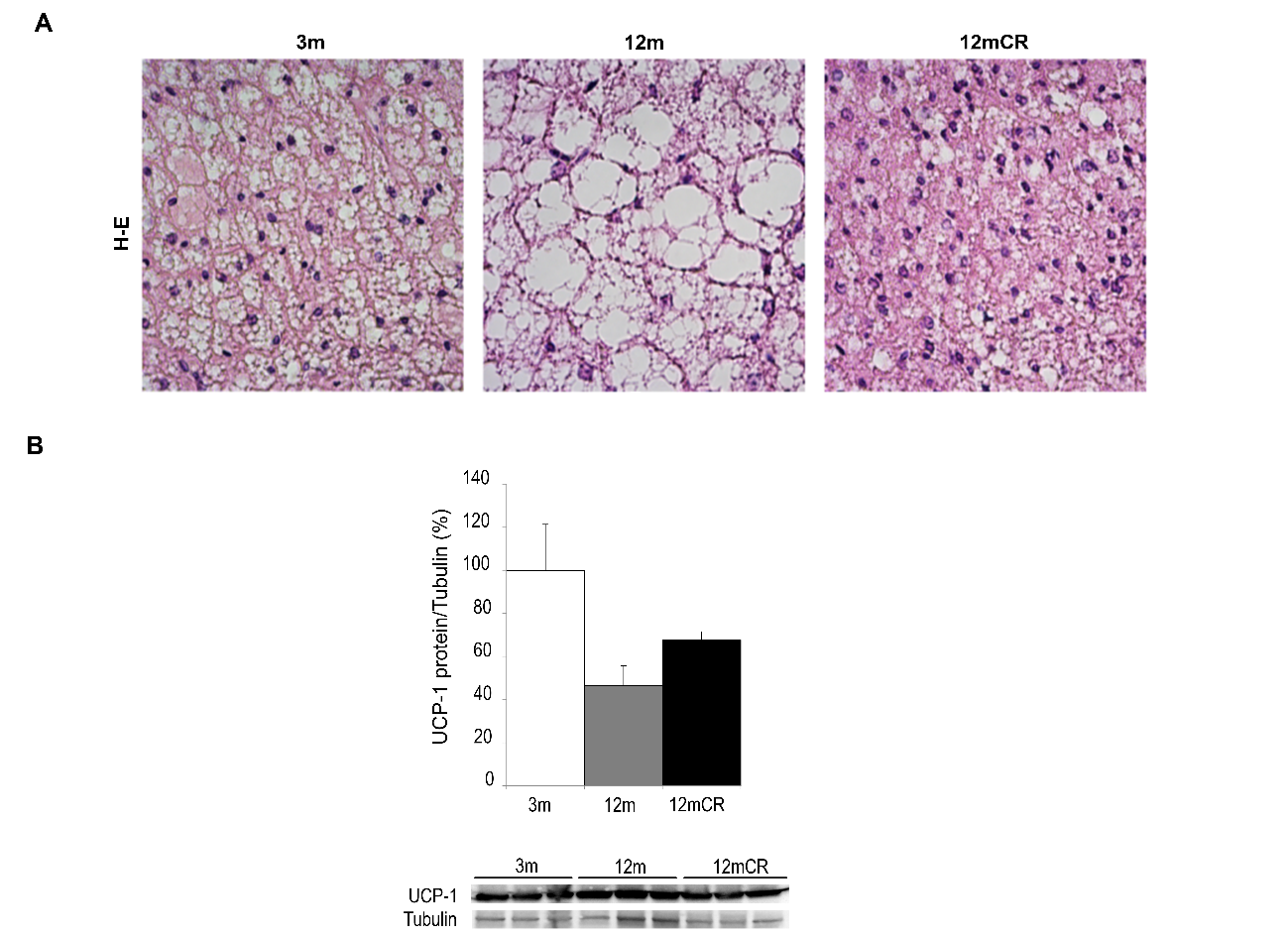


**Figure S4. Morphology and protein changes in BAT at middle-age.** (A) Representative images of H-E staining in paraffin-embedded BAT histological sections (magnification 40×, scale bar = 20μm) of the experimental groups (*n* = 4 animals/group). (B) Quantification of UCP-1 total protein expression in BAT. Levels of protein were normalized to total Tubulin. All data are expressed as mean ± SEM (*n* = 4 animals/group).


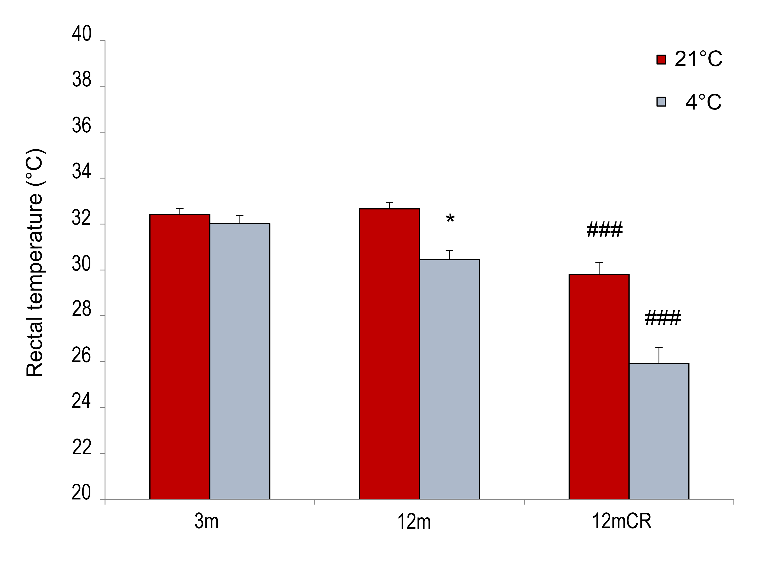


**Figure S5. Rectal temperature before and after cold exposure.** Rectal temperature was measured in 3m, 12m and 12mCR mice before and after 24 h of exposure to a 4°C environment. Data are expressed as mean ± SEM (*n* = 7–9 animals/group). ^*^*P* <0.05, 12m *vs.* 3m; ^###^*P*<0.001, 12mCR *vs.*12m.
